# Physical Exercise for Health and Performance Post-Pandemic COVID-19 Era, a Renewed Emphasis on Public Health

**DOI:** 10.3390/ijerph19116475

**Published:** 2022-05-26

**Authors:** Iván Chulvi-Medrano, Ewan Thomas, Elvira Padua

**Affiliations:** 1 Sport Performance and Physical Fitness Research Group (UIRFIDE), Department of Physical and Sports Education, University of Valencia, 46010 Valencia, Spain; 2 Sport and Exercise Research Unit, Department of Psychological, Pedagogical and Educational Sciences, University of Palermo, 90146 Palermo, Italy; ewan.thomas@unipa.it; 3 Department of Human Sciences and Promotion of the Quality of Life, San Raffaele Roma Open University, 00166 Rome, Italy; elvira.padua@uniroma5.it

After the period of forced quarantine due to the COVID-19 epidemic, the physiological principle of detraining became more evident than ever. Reducing the amount of physical exercise has been shown to have negative effects on health [1,2] and athletic/sports performance [3].

In this sense, physical exercise can attenuate this decline [4,5]. Physical exercise as a stressful stimulus responds to a dose–response relationship in which there must be a minimum of physical exercise to trigger/induce adaptations [6,7], always taking into account the interindividuality of responses and adaptations [8,9]. An example is represented in a recent meta-analysis in which, after analyzing seven prospective cohort studies including 175,370 people, an inverse linear association between the number of daily steps from 2700 to 17,000 and the risk of mortality was found [10].

On the relationship between exercise and health, it is of great interest to provide data on the beneficial effects of physical exercise on health and quality of life in pediatric [11,12], adult [13,14] and elderly [15,16] stages.

In the first place, the benefit of physical exercise is established by the increase in the daily energy cost, which prevent and even reverse the risk of the Sedentary Death Syndrome [1]. Therefore, physical exercise represents a non-pharmacological medicine that should be administered as primary prevention for the main 35 chronic diseases [17]. In addition, it is known that physical exercise plays an important role in tertiary prevention by providing a non-pharmacological strategy for the management of different established pathologies [18].

In relation to the psychosocial domain of health, the benefits of physical exercise were well known [19]. However, due to the pandemic, there has been increased concern about mental health and how physical exercise can positively influence [20,21,22]. It has been recently shown that even below minimum levels of physical activity can have a protective effect against depression [22].

Sports science oriented to physical performance in sports, in recent years, has highlighted the importance of paying attention to factors other than training, such as nutrition [23,24] and sleep/recovery [25,26,27]. These factors have a great influence on physical performance [28]. Strategies that optimize recovery will have restorative effects on physiological and cognitive systems, allowing adaptations and a decrease in the risk of fatigue-induced injury [24].

In recent years, sports science has deepened in the study of molecular biology, which allows understanding the physiological processes that would explain the exercise-induced effects and/or benefits expected for both health and performance. Thus, for example, since the discovery in 2000 of the myokine IL-6 [29] and its benefits, much progress has been made, allowing the coining of the concept of exerkinines [30]. As a result, findings have shown that physical exercise favors cross-talk between muscle and various tissues such as bone [31].

For all these reasons, the need to deepen the knowledge provided by sports sciences is evident. Therefore, the idea of this Special Issue is to call for the submission of articles that allow a multidisciplinary approach for the optimization of health and physical-sports performance.
